# Analysis of Phenol Biodegradation in Antibiotic and Heavy Metal Resistant *Acinetobacter lwoffii* NL1

**DOI:** 10.3389/fmicb.2021.725755

**Published:** 2021-09-10

**Authors:** Nan Xu, Chong Qiu, Qiyuan Yang, Yunzeng Zhang, Mingqi Wang, Chao Ye, Minliang Guo

**Affiliations:** ^1^College of Bioscience and Biotechnology, Yangzhou University, Yangzhou, China; ^2^School of Food Science and Pharmaceutical Engineering, Nanjing Normal University, Nanjing, China

**Keywords:** *Acinetobacter lwoffii*, Phenol, bioremediation, multidrug resistance, heavy metals

## Abstract

Phenol is a common environmental contaminant. The purpose of this study was to isolate phenol-degrading microorganisms from wastewater in the sections of the Chinese Medicine Manufactory. The phenol-degrading *Acinetobacter lwoffii* NL1 was identified based on a combination of biochemical characteristics and 16S rRNA genes. To analyze the molecular mechanism, the whole genome of *A*. *lwoffii* NL1 was sequenced, yielding 3499 genes on one circular chromosome and three plasmids. Enzyme activity analysis showed that *A*. *lwoffii* NL1 degraded phenol *via* the *ortho*-cleavage rather than the *meta*-cleavage pathway. Key genes encoding phenol hydroxylase and catechol 1,2-dioxygenase were located on a megaplasmid (pNL1) and were found to be separated by mobile genetic elements; their function was validated by heterologous expression in *Escherichia coli* and quantitative real-time PCR. *A*. *lwoffii* NL1 could degrade 0.5 g/L phenol within 12 h and tolerate a maximum of 1.1 g/L phenol, and showed resistance against multiple antibiotics and heavy metal ions. Overall, this study shows that *A*. *lwoffii* NL1 can be potentially used for efficient phenol degradation in heavy metal wastewater treatment.

## Introduction

Phenolic contaminants have been recently caused by rapid urbanization and industrialization. Phenol and phenolic compounds are usually discharged from various wastewater of petrochemical, pharmaceutical, and chemical processing industries (Ha et al., 2000; Duan et al., 2018). The concentration of volatile phenol in drinking water should be below 0.001 mg/L, and the maximum allowable concentration in the source water is 0.002 mg/L established by the World Health Organization (Paisio et al., 2014). Excessive phenol hinders the growth of animals and plants in the polluted environment and can even cause their death. In addition, phenol is easily converted into deleterious aromatic compounds by reacting with chlorine gas or iron ions, yielding chlorophenol or phloroglucinol (Pankaj and Hanhong, 2014). Because of the toxic effects of phenol and its degradation products, this compound has been categorized as a priority hazardous pollutant. The removal of phenol from polluted water depends on physical, chemical, and biological methods. The biological methods are mainly based on the application of microorganisms, which can transform phenol to harmless low-carbon compounds by their own metabolic system. The sustainable, efficient, and cost-effective cleaning technology has received increasing attention regarding the treatment of phenol-polluted environments (Rucka et al., 2017; Chandrasekaran et al., 2018).

The bacteria of the genus *Pseudomonas* have been used as typical phenol-degrading microorganisms. *P. putida* can degrade 1 g/L phenol in 162 h (6.17 mg/L per hour) (Kumar et al., 2005), whereas *P. cepacia* isolated from industrial wastewaters can degrade 2.5 g/L phenol in 144 h (17.36 mg/L per hour) (Arutchelvan et al., 2005). *Acinetobacter calcoaceticus* can degrade 91.6% of 0.8 g/L phenol in 48 h (15.27 mg/L per hour) (Liu et al., 2016). A new *Rhodococcus aetherivorans* strain has the degradation rates of 35.7 mg/L per hour at 0.5 g/L phenol (Nogina et al., 2020). The mutants M1 of *Rhodococcus ruber* SD3 can degrade 98% of 2 g/L phenol in 72 h by cell immobilization (27.2 mg/L per hour) (Peng et al., 2013). In addition, some yeasts and fungi have also been used for phenol degradation. *Candida tropicalis*, the model phenol-degrading yeast, could decompose 2.6 g/L phenol within 70.5 h after He–Ne laser mutation (36.88 mg/L per hour) (Jiang et al., 2007). Phenol-degrading filamentous fungi mainly include *Aspergillus oryzae* and *Aspergillus flavus* (Ghanem et al., 2009; Krivobok et al., 1994). To improve the efficiency of phenol degradation by microorganisms, several approaches such as selection in a heavily phenol-polluted environment, mutagenesis, and immobilization of microbial cells have been employed.

The biodegradation of phenol by microorganisms can occur in aerobic and anaerobic conditions (Gao et al., 2010; Tomei et al., 2021). Activated sludge is at present widely used as a type of aeration-based wastewater treatment. The biochemical process of phenol degradation by aerobic microorganisms includes the conversion of complex aromatic metabolites to primary C3-C4 compounds necessary for bacterial growth. Phenol is first oxidized by phenol hydroxylase into catechol. The catechol is then transformed *via* various ring-opening reactions, including *ortho*-cleavage and *meta*-cleavage catalyzed by catechol 1,2-dioxygenase and catechol 2,3-dioxygenase, respectively. In the *ortho*-cleavage pathway, catechol is transformed into cis,*cis*-muconate and then into succinyl-CoA (MacLean et al., 2006), whereas in the *meta*-cleavage pathway, catechol is converted into 2-hydroxymuconate semialdehyde, then into 2-keto-4-pentenoic acid *via* two mechanisms, and finally into acetyl-CoA (Kukor and Olsen, 1991).

Although the pathways for aerobic phenol decomposition are established and many aerobic phenol-degrading microorganisms have been isolated, the search for bacterial strains effectively removing phenol in specific environments is ongoing. In this study, one kind of highly efficient phenol-degrading microorganism was gained from slightly polluted wastewater in the Yangzhou Grand Canal, and was identified as *Acinetobacter lwoffii* NL1. Its degradation capacity and pathway were investigated. *A. lwoffii* is a Gram-negative bacterium of the *Acinetobacter* genus belonging to the class Gammaproteobacteria. Previous studies have mainly focused on the isolation of clinical strains, their pathogenicity, and the treatment of drug-resistant isolates (Mlynarcik et al., 2019; Rathinavelu et al., 2003). Few reports indicate that *A. lwoffii* strains DNS32, C1, and ISP4 can degrade aromatic pollutants such as atrazine (Zhang et al., 2012), simazine (Sacca et al., 2012) and isophthalate (Vamsee-Krishna and Phale, 2010), respectively. Isolate BDCC-TUSA-12 from a refinery wastewater plant, which is highly similar to *A. lwoffii* JCM 6840, was reported to utilize 0.5 g/L phenol in 7 days (Bahobail et al., 2016). In the present study, *A. lwoffii* NL1 completely degraded 0.5 g/L phenol in 12 h without any strain modification or process optimization. Such degradation efficiency (41.67 mg/L per hour) is higher than that of other reported microorganisms. To elucidate the molecular mechanism underlying phenol degradation by *A. lwoffii* NL1, its whole genome was sequenced and the genes predicted by genome annotation to be associated with the degradation pathway were tested by the transcriptional response to phenol induction. The catalytic activity of the encoded proteins was confirmed by heterologous expression in *Escherichia coli*. Another characteristics of drug and heavy metals resistance were identified. In this study, *A. lwoffii* NL1 was comprehensive analyzed by combining biochemical, genomic, and genetic methods.

## Materials and Methods

### Strains

A phenol-degrading strain *A. lwoffii* NL1 was isolated in this study and preserved in the China Center for Type Culture Collection (CCTCC NO: M2014329). The *E. coli* strains DH5α and BL21 were purchased from Invitrogen.

### Isolation of Phenol-Degrading Strains

Wastewater samples were collected on the southern sections of the Chinese Medicine Manufactory along the Yangzhou Grand Canal, Yangzhou City, Jiangsu Province, China. Samples were placed on crushed ice, and then gradiently diluted on 0.05 g/L ∼0.2 g/L phenol-containing Luria Broth (LB) agar plates for 48 h at 30°C. The colony-forming isolates were further purified by growth on the plates. The enriched single colonies were inoculated in 50 mL of sterile liquid minimal mineral (MM) medium (NH_4_Cl, 1.0 g/L; NaH_2_PO_4_, 1.0 g/L; K_2_HPO_4_, 3.0 g/L; KCl, 0.15 g/L; MgSO_4_⋅7H_2_O, 0.3 g/L; CaCl_2_, 0.01 g/L; FeSO_4_⋅7H_2_O, 2.5 mg/L; pH 7.0) containing filter-sterilized 0.1 g/L phenol. The biomass of the isolates was compared at 28°C for 36 h with agitation (200 rpm), and the fastest growing strain was selected.

### Strain Identification

Traditional strain identification was performed after isolating phenol-degrading strains. Microbial characteristics were determined using biochemical identification tubes (Hangzhou Microbial Reagent, China). Sequences of 16S rRNA genes were amplified using bacterial universal primers and compared with those of 16S rRNA genes from other *Acinetobacter* species. MEGA X software was used to construct phylogenetic trees by the Neighbor-joining method (Kumar et al., 2018).

Genome-based taxonomy was done after sequencing the genome of the strain NL1. The genome sequences of six *Acinetobacter pseudolwoffii* strains, ten *Acinetobacter lwoffii* strains, and the strain NL1 were submitted into Type (Strain) Genome Server^1^. Evolutionary relationships between 17 strains were inferred by the Genome BLAST Distance Phylogeny method (Meier-Kolthoff et al., 2013). Genome sequences of *A. pseudolwoffii* (ANC 5044^*T*^, ANC 5318, ANC 5324, ANC 5347, NIPH 713, F78) and *A. lwoffii* (ANC 4400, NIPH 512^*T*^, NIPH 715, NIPH 478, CIP A162, CIP 64.7, CIP 51.11, CIP_101966, CIP 102136, CIP 70.31) were downloaded from www.ncbi.nlm.nih.gov/genome.

### Evaluation of Phenol Degradation

Cultures grown in LB test tubes were gradiently diluted from 0.1 to 10^–6^. Then 5 μL cultures were spotted onto MM plates (1.5% agar) containing phenol at concentrations ranging from 0 to 1.1 g/L. The growth phenotype of *A. lwoffii* NL1 was observed after 2 days to assess phenol tolerance.

Phenol degradation under different culture conditions was evaluated in liquid MM medium with phenol as the sole carbon source. Single colonies were first activated in LB test tubes at 28°C and 200 rpm for 36 h. Cells were washed and appropriately diluted using sterile K_3_PO_4_ buffer and then incubated in 50 mL of MM medium with phenol as the sole carbon source. Cultivation parameters, including initial phenol concentration (0, 0.2, 0.5, 0.6, and 0.7 g/L), inoculum volume (v/v, 2%, 5%, 8%, 10%, 12%, and 15%), initial pH (5, 6, 7, 8, 9, and 10), and temperature (25°C, 38°C, 30°C, 33°C, 35°C, 37°C, and 40°C) were analyzed separately to observe the influence on cell growth and phenol utilization in strain NL1. Bacterial growth was assessed by the optical density (OD) of biomass measured at 600 nm using a spectrophotometer, and phenol concentrations were monitored by the colorimetric assay using 4-aminoantipyrine (King et al., 1991). The results were expressed as the means standard error of the mean from three repetitions using the Microsoft Office Excel’s data analysis tool (2019 version).

### Genome Sequencing and Gene Annotation

High-quality genomic DNA of *A*. *lwoffii* NL1 was obtained using the Rapid Bacterial Genomic DNA Isolation Kit (Sangon Biotech, China) and analyzed using a NanoDrop spectrophotometer and a Qubit fluorometer. Subsequently, each library was assessed for quantity and size and sequenced on a PacBio Sequel platform. Raw sequence reads were introduced into the non-hybrid Hierarchical Genome Assembly Process (HGAP version 4). Repeat sequences in replicons were marked using RepeatModeler and RepeatMasker (Chen, 2004). The location and sequences of genes and proteins were predicted by the Prodigal software (Hyatt et al., 2010). The rRNA and tRNA genes were identified using the barrnap and tRNAscan software, respectively (Lowe and Eddy, 1997). Functions of putative genes were annotated using the Clusters of Orthologous Groups of proteins (COG) (Tatusov et al., 1997) and Gene Ontology (GO) annotation (Pillai et al., 2012). NCBI BLAST was used for annotating genes encoding phenol hydroxylase. The megaplasmid pNL1 was characterized using the RAST annotation server (Aziz et al., 2008). Resistance genes were predicted using a combination of the Comprehensive Antibiotic Resistance Database (CARD) (Alcock et al., 2020) and the Center for Genomic Epidemiology (CGE) tools^2^. Mobile elements such as insertions and integrons were annotated using ISfinder^3^ and INTEGRALL (Moura et al., 2009), respectively.

### Construction of *E. coli* Expression Mutants

The genes encoding phenol hydroxylase (*LSNL_2975–2980*) and catechol 1,2-dioxygenase (*LSNL_2981*) of *A*. *lwoffii* NL1 were heterologously expressed in *E. coli*. The gene cluster *LSNL_2975–2980* and the gene *LSNL_2981* were amplified using primer pairs PH-F/PH-R and 1,2-CTD-F/1,2-CTD-R, respectively (Table 1), and diluted bacterial culture as a template. The target fragments were identified by agarose gel electrophoresis (1%) and recovered using the TaKaRa Agarose Gel DNA Extraction Kit (TaKaRa, Japan). The multicopy expression vector pET-30a (Novagen) was digested with *Eco*RI and *Xho*I, and ligated with the target PCR fragments using ligation-free cloning Master Mix (Abmgood, Canada), yielding pET-30a-PH and pET-30a-1,2-CTD, respectively. The constructed plasmids were then used to transform *E. coli* BL21. Mutants were selected and validated using the kanamycin resistance gene as a positive selection marker.

**TABLE 1:** Primers used in this study.

Primer	Sequences (5′ to 3′)
PH-F	ATCGGATCCGAATTCATGAAGGATGCAACAGGC
PH-R	GTGGTGGTGCTCGAGTTAAATATGTTTAAACAATGCAG
1,2-CTD-F	ATCGGATCCGAATTCATGGACCGTCAACAAATTGAT
1,2-CTD-R	GTGGTGGTGCTCGAGTTATGTGCTTGCGCGGC
16srRNA-F	CTCGCAGAATAAGCACCG
16srRNA-R	CTCCCATACTCTAGCCAACC
LSNL_2975-F	TTTTGTGCCACCAATCAG
LSNL_2975-R	ACGCCATAACGCCATTT
LSNL_2976-F	TCCGCTGTGGAAACCTG
LSNL_2976-R	GATATGACGAAACGGC
LSNL_2977-F	AAGAAGACAACCCAGATGC
LSNL_2977-R	TTGCCACCCAGCGTAAT
LSNL_2978-F	CAAGCGTTTCTACAGCG
LSNL_2978-R	TTCCAAATCTAAGCCACC
LSNL_2979-F	GCTATTCGCCCAGATTACG
LSNL_2979-R	CGTGTGCAGAACAAT
LSNL_2980-F	TTTCAGGCAGGGCAGT
LSNL_2980-R	CCCGATTCAAGCAGGTC
LSNL_2981-F	TAACCGCCCATCTCACG
LSNL_2981-R	TTCACGGGTAGCAAAGG

### Enzymatic Activity

Key enzymes for the phenol metabolic pathway, phenol hydroxylase, catechol 1,2-dioxygenase, and catechol 2,3-dioxygenase were analyzed in strain NL1 and recombinant *E. coli* strains. *A. lwoffii* NL1 was cultivated in MM medium containing 0.5 g/L phenol. Two types of *E. coli* expression mutants were grown in LB medium supplemented with 50 μg/L kanamycin. When cultures reached OD_600_ 0.5–0.6, cells were collected by centrifugation, resuspended in 0.1 mol/L K_3_PO_4_ buffer, subjected to sonic disruption of up to 15 min with intervals, and centrifuged at 12,800 *g* for 10 min at 4°C. The obtained supernatants were used as crude enzyme samples.

Total protein concentration was quantified using the Brilliant Blue method (Bradford, 1976). Considering NADPH as one substrate of phenol hydroxylases, phenol hydroxylase activity could be expressed as a decrease of NADPH. The phenol hydroxylase activity was measured using a modified Neujahr’s method (Neujahr and Kjellén, 1980). The 200-μL enzymatic reaction mixture containing 0.2 mM FAD, 0.1 mM NADPH, 1 mM phenol, and 0.1 M K_3_PO_4_ buffer (pH 7.5), and 50 μL crude enzyme was incubated at 30°C and the absorbance (A) at 340 nm was read every 30 s. The above 200-μL enzymatic reaction mixture without phenol was the control group of phenol hydroxylase activity in crude enzymes extracts. The activities of catechol 1,2-dioxygenase and catechol 2,3-dioxygenase were determined at 25°C based on the production of cis,*cis*-muconic acid and 2-hydroxymuconate semialdehyde measured at A_260_ and A_375_, respectively (Ngai et al., 1990; Sala-Trepat and Evans, 1971). One unit of enzyme activity was defined as the amount of enzyme that consumed or generated 1 μM substrate or product, respectively, per minute, and specific activity was defined as the number of units per milligram of protein.

### Transcriptional Analysis

*A*. *lwoffii* NL1 suspension was transferred to MM medium containing 16 mM sodium acetate; 2 mM phenol was added to experimental cultures, whereas control cultures were grown without phenol. Log-phase bacteria (OD_600_ 0.5–0.6) were washed with PBS (pH 7.4) and digested with 400 μg/mL lysozyme for 4 min. Total RNA was isolated using chloroform, ethanol, and the Bacteria Total RNA Isolation Kit (Sangon Biotech, China); cDNA was synthesized from 35 ng RNA using the PrimeScript^TM^ RT Reagent Kit with gDNA Eraser (TaKaRa) and stored at −20°C. The expression of the target genes (*LSNL_2975–2981*) was first detected by PCR using primer pairs targeting 20-bp regions flanking the coding sequence (CDS). Then, gene expression levels were quantified by quantitative real-time PCR (qRT-PCR) in the CFX Connect Real-Time PCR Detection System (Bio-Rad, United States). A 20 μL qRT-PCR mixture contained 10 μL SYBR^®^ Premix Ex Taq^TM^ II (2×), 0.8 μL of each forward and reverse primer (Table 1, LSNL_2975∼2981-F/R), 0.4 μL ROX Reference Dye II (50×), 2 μL cDNA sample (50 ng/μL) as a template, and 6 μL double-distilled H_2_O. The expression of the target genes was normalized to those of the 16S rRNA genes, and relative expression levels were determined using the 2^–ΔΔ*Ct*^ method.

### Antibiotics Susceptibility and Heavy Metals Testing

*A. lwoffii* NL1 cultures grown overnight were diluted to OD_600_ = 0.1 using 0.9% NaCl solution and spread on LB or metal ion-containing LB plates. The antibiotic sensitivity of *A. lwoffii* NL1 was determined by the disk diffusion method. Paper disks impregnated with 30 different antibiotics, including erythromycin, minocycline, cefuroxime, cefalexin, ciprofloxacin, norfloxacin, ceftriaxone, and penicillin were placed on the LB plate surface, and the diameters of inhibition zones were measured after 48 h. Susceptibility results successively follow the table 2B-2 in the M100 performance standards for antimicrobial susceptibility testing from CLSI (Clinical & Laboratory Standard Institute), the Table 3 in the standard of antibiotics susceptibility test (Kirby-Bauer method), and the instruction of drug disks. Specific standards for 30 tested antibiotics were listed in Supplementary Table 1. The used salts (mM) in the metal ion-containing LB plates (Mindlin et al., 2016) included: H_2_O⋅Fe_2_(SO_4_)_3:_ 0.4, 0.8, 1.6, 2.0, and 3.2; CuSO_4_⋅5 H_2_O: 0.9, 1.8, 2.7, 3.6, and 4.0; AgNO_3:_ 0.01, 0.05, and 0.1; NiSO_4_⋅6H_2_O: 0.45, 0.9, 1.8, and 2.7; CoCl_2_ × 6H_2_O (Co): 0.01, 0.05, and 0.1; ZnCl_2_: 0.2, 0.4, 0.8, 1.6, 3.2, and 6.4; HgCl_2_ (Hg): 0.015, 0.03, 0.045, and 0.06. The plates were visually inspected after 24 h.

**TABLE 2:** Homology comparison of *A. lwoffii* NL1 phenol hydroxylase-encoding genes.

Locus Tag	Homologous gene	Bacterium	Identity	Function
LSNL_2975	dmpK aphK mphK	*Pseudomonas* sp.CF600 *Comamonas testosteroni* TA441 *Acinetobacter calcoaceticus* PHEA-2	54.1% 43.2% 69.0%	Auxiliary protein
LSNL_2976	dmpL aphL mphL	*Pseudomonas* sp.CF600 *Comamonas testosteroni* TA441 *Acinetobacter calcoaceticus* PHEA-2	48.8% 41.4% 79.8%	Phenol hydroxylase large subunit
LSNL_2977	dmpM aphM mphM	*Pseudomonas* sp.CF600 *Comamonas testosteroni* TA441 *Acinetobacter calcoaceticus* PHEA-2	57.0% 40% 89.8%	Phenol hydroxylase activator protein
LSNL_2978	dmpN aphN mphN	*Pseudomonas* sp.CF600 *Comamonas testosteroni* TA441 *Acinetobacter calcoaceticus* PHEA-2	71.4% 65.4% 92.9%	Phenol hydroxylase large subunit
LSNL_2979	dmpO aphO mphO	*Pseudomonas* sp.CF600 *Comamonas testosteroni* TA441 *Acinetobacter calcoaceticus* PHEA-2	41.5% 32.8% 66.7%	Phenol hydroxylase small subunit
LSNL_2980	dmpP aphP mphP	*Pseudomonas* sp.CF600 *Comamonas testosteroni* TA441 *Acinetobacter calcoaceticus* PHEA-2	68.8% 54.2% 89.5%	FAD-containing reductase component

**TABLE 3:** Antimicrobial susceptibility of *A. lwoffii* NL1.

Antibiotics	Disk content (μg)	Zone of inhibition (mm)	Results
Chloramphenicol	30	29	S
Tetracycline	30	14	I
Kanamycin	30	0	R
Clindamycin	2	11	R
Erythromycin	15	17	I
Ceftazidime	30	0	R
Minocycline	30	17	R
Cefuroxime	30	16	I
Doxycycline	30	21	S
Furazolidone	300	12	R
Cefradine	30	13	R
Cefazolin	30	13	R
Selectrin	23.75	8	R
Polymyxin B	300	13	S
Neomycin	30	19	S
Cefalexin	30	15	I
Vancomycin	30	11	I
Piperacillin	100	11	R
Ciprofloxacin	5	17	I
Gentamicin	10	21	S
Carbenicillin	100	18	R
Ofloxacin	5	17	S
Amikacin	30	22	S
Ampicillin	10	17	I
Norfloxacin	10	13	I
Medemycin	30	9	R
Cefoperazone	75	15	R
Oxacillin	1	0	R
Ceftriaxone	30	20	I
Penicillin	100	18	I

*S, susceptible; I, intermediate; R, resistance.*

## Results

### Screening and Identification of Bacteria

**FIGURE 1:**
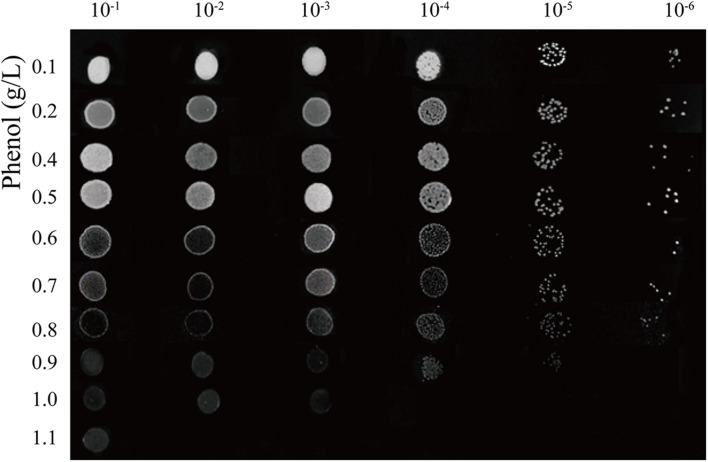
Pot assays of phenol tolerability of *A. lwoffii* NL1.

Wastewater samples were collected from the southern sections of the Chinese Medicine Manufactory along the Yangzhou Grand Canal. 12 isolates from 15 samples could tolerate 0.2 g/L phenol on LB agar plates were chosen for the secondary screening. Then only 3 isolates could grow on 0.1 g/L phenol-containing medium, and strain NL1 show the strongest phenol-degrading activity. Analysis of strain NL1 phenol tolerability on MM plates with an increased phenol concentration revealed that colony density increased with phenol concentration from 0 to 0.5 g/L (Figure 1), which demonstrated that phenol could support bacterial growth as a carbon source. Cell growth was gradually inhibited by higher phenol concentrations, and the maximum concentration tolerated by strain NL1 was 1.1 g/L. NL1 colonies were round, milky white, opaque, and slightly moist, and could reach a diameter of about 1–1.5 mm after growing on LB plates for 2 days. Morphological analysis by optical microscopy revealed that NL1 cells were short rods of 2.5–2.9 μm- long by 1.8–2.1 μm-wide (Supplementary Figure 1). Strain NL1 was oxidase- and catalase-negative and could not ferment glucose, lactose, xylose, maltose, and sucrose. In addition, the strain was negative for esculin hydrolysis, H_2_S production, and sodium citrate utilization but positive in lysine and ornithine tests. Overall, biochemical tube test results were in accordance with the general characteristics of *Acinetobacter*. strain NL1 was further identified by 16S rRNA sequencing. The partial sequence of the 16S rRNA gene was a continuous stretch of 1345 bp, which exhibited 99.7% identity and zero branch length compared to the *A. lwoffii* JCM 6840 gene (Supplementary Figure 2). Based on these results, strain NL1 was identified as *A. lwoffii* and was preserved in the China Center for Type Culture Collection (CCTCC NO: M20191004).

### Sequencing and Annotation of the *A. lwoffii* NL1 Genome

**FIGURE 2:**
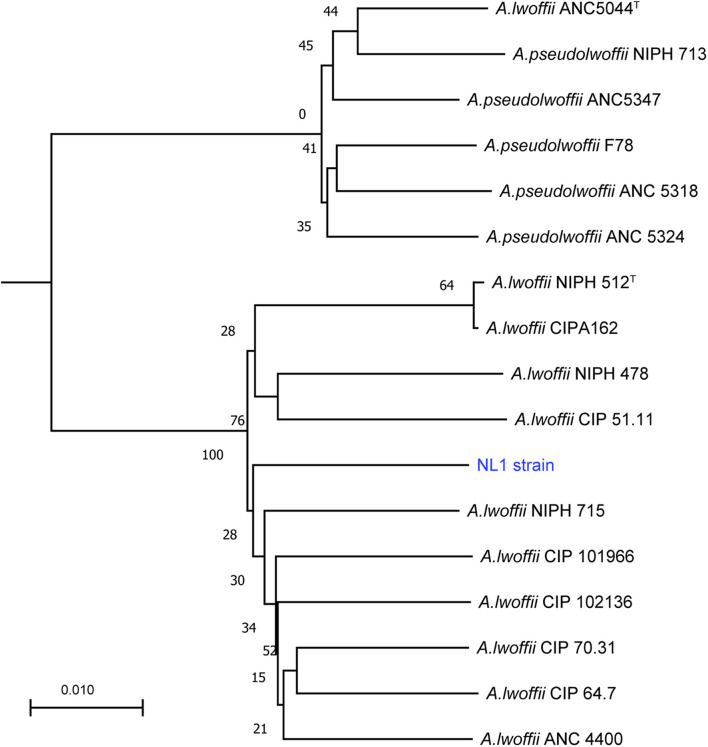
Whole-genome sequence-based phylogenetic tree of *A. lwoffii* and *A. pseudolwoffii* strains.

We sequenced the genome of *A. lwoffii* NL1 on a PacBio Sequel platform. Four polished contigs with a mean length of 20,551 nt were *de novo* assembled from quality control-filtered 52,660 subreads using the HGAP4 analysis application (SMRT Link 4.0) (Chin et al., 2013). Taxonomy of the *Acinetobacter lwoffii* group had been revised including two monophyletic subbranches (Nemec et al., 2019). Using genome-based methods (Meier-Kolthoff and Göker, 2019), the strain NL1 was classified into the *lwoffii* species not into *pseudolvoffii* (Figure 2). Genomic features of *A. lwoffii* NL1 were in the Supplementary Table 2. The genome of *A. lwoffii* NL1 contained 3,661,075 bp and its GC content was 43.1%. A 3,116,590-bp circular chromosome represented 85.13% of the whole genome. Three extra-chromosomal elements were found in *A. lwoffii* NL1: two megaplasmids, pNL1 (317 kb) and pNL2 (189 kb), and a smaller plasmid (36 kb). The 3499 protein-coding sequences predicted in the genome corresponded to 85.6% of the total CDS length; 86 tRNA and 21 rRNA genes were also identified. The 3499 protein-coding genes were functionally annotated: 2717 were classified with GO and 2226 with COG. There were 2.02% of repeat sequences in the NL1 genome, and they included 120 unclassified interspersed repeats (67,867 bp), 92 simple repeats (4479 bp), and low complexity repeats (1766 bp), whereas no CRISPR-like repeats were found. Sequence data of the chromosome and three plasmids of *A. lwoffii* CCTCC M20191004 have been deposited at GenBank under the accession number CP062199-CP062122. Then, a genome-scale search and comparative analysis with sequences of other known phenol-degrading microorganisms were performed to identify *A. lwoffii* NL1 genes related to phenol degradation.

### Genes Annotated for Phenol Degradation

**FIGURE 3:**
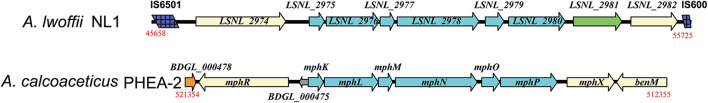
Structures of phenol-degrading genes in *A. lwoffii* NL1 and *A. calcoaceticus* PHEA-2. The gene cluster *LSNL_2973-2983* is located on megaplasmid pNL1 (between 45,658 and 55,725) in *A. lwoffii* NL1. The gene cluster *BDGL_000467-000478* is positioned on the chromosome (between 512355 and 521354) of *A. calcoaceticus* PHEA-2. Cyan and green arrows represent genes for phenol hydroxylase and catechol 1,2-dioxygenase, respectively. Transcriptional regulators are present yellow arrows. *BDGL_000475* and *BDGL_000478*, respectively, encode hypothetical protein and copper chaperone in *A. calcoaceticus* PHEA-2. Putative transposases for insertion sequence elements are represented using quadrilateral meshes in *A. lwoffii* NL1.

Phenol hydroxylase, the first enzyme in the phenol degradation process, can be monomeric, two-component, and multi-component. Monomeric phenol hydroxylases are mainly found in eukaryotic yeast and only in a few bacteria such as *Pseudomonas pickettii* and *Corynebacterium glutamicum* (Kukor and Olsen, 1992; Shen et al., 2005). Two-component phenol hydroxylases, with the function of oxygenase and reductase, were encoded by two adjacent genes such as *PheA1* and *PheA2* in *Rhodococcus erythropolis* UPV-1 (Laura et al., 2010), *Rhodococcus opacus* 1CP (Eulberg et al., 1998), and *Geobacillus stearothermophilus* (Omokoko et al., 2008). Multi-component phenol hydroxylases (monooxygenases), which are widely represented in bacteria, have environmental advantages. *A. lwoffii* NL1 had six genes encoding phenol hydroxylase located in tandem on the circular megaplasmid pNL1 with 39.2% G + C content. Phenol hydroxylase in *A. lwoffii* NL1 showed a high degree of sequence conservation when compared with other multi-component enzymes (Table 2). These genes (*LSNL_2975–2980*) had 69%, 80%, 90%, 93%, 67%, and 90% identity with *mphK*, *mphL*, *mphM*, *mphN*, *mphO*, and *mphP* of *A. calcoaceticus* PHEA-2, respectively (Xu et al., 2003). As shown in Figure 3, the structure of related phenol-degrading genes of *A. lwoffii* NL1 (*LSNL_2973-2983*) was compared with that of *A. calcoaceticus* PHEA-2 (*BDGL_000467-000478*). In spite of the aforementioned similarity, the *LSNL_2981* encoding catechol 1,2-dioxygenase was not found a homologous gene on the phenol degradation locus of *A. calcoaceticus* PHEA-2. The *LSNL_2973* and *LSNL_2983* located upstream and downstream of phenol degradation operon were putative transposases for insertion elements in *A. lwoffii* NL1. Genes *BDGL_000478* and *BDGL_000468* positioned before and after *mphR* and *mphX* in *A. calcoaceticus* PHEA-2 were putative copper chaperone and LysR family regulatory protein, respectively. Different from three gene sets of two-component phenol hydroxylase in *Rhodococcus* (Gröning et al., 2014), one gene set encoding phenol hydroxylase was found in the megaplasmid pNL1 of *A. lwoffii* NL1. These genes (*LSNL_2975–2980*) of *A. lwoffii* NL1 were compared with 31 genomes of all the reported *Acinetobacter lwoffii* strains in NCBI Genome database. No homologous alignments reflected the uniqueness of *A. lwoffii* NL1.

The *LSNL_2981* gene annotated to encode catechol 1,2-dioxygenase, the second key enzyme of phenol *ortho*-cleavage, was located in the plasmid after the phenol hydroxylase genes. However, no catechol 2,3-dioxygenase genes were identified in *A. lwoffii* NL1 by genome-scale search. It should be noted that genes *LSNL_2974* and *LSNL_2982* exhibited similarity with *A. calcoaceticus* PHEA-2 *mphR* (80%) and *mphX* (67.6%), respectively, which were suggested to be involved in the transcriptional regulation of phenol hydroxylase (Yu et al., 2011).

### Validation of Genes Related to Phenol Catabolism

Catabolism of phenol, including production of secondary and primary metabolites, varies depending on microbial species. As an aerobic Gram-negative bacterium, *A. lwoffii* NL1 should first oxidize phenol to catechol through activity of phenol hydroxylase. In crude extracts of NL1 cells, the enzymatic activity of phenol hydroxylase was 0.13 U/mg. About the pathway of catechol conversion in strain NL1, we also measured activities of catechol 1,2-dioxygenase and catechol 2,3-dioxygenase. The activity of catechol 1,2-dioxygenase was 1.48 U/mg but that of catechol 2,3-dioxygenase was not detected. Results on enzymatic activity were consistent with gene annotation, confirming that *A. lwoffii* NL1 degraded phenol through the ortho- but not meta-cleavage pathway.

**FIGURE 4:**
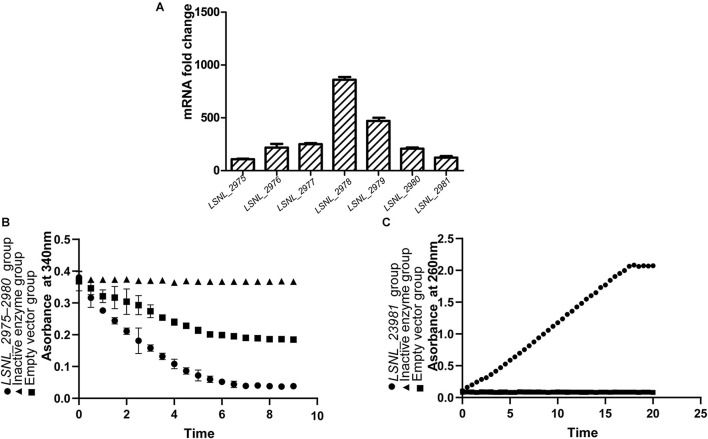
Gene expression and catalytic activity of phenol degradation-related enzymes. **(A)** mRNA expression folds of *LSNL_2975–2981* genes in *A. lwoffii* NL1 with phenol compared to cultures without phenol. **(B,C)** Phenol hydroxylase **(B)** and catechol 1,2-dioxygenase **(C)** expressed in *E. coli* BL21.

Gene expression of phenol metabolism-related genes in *A. lwoffii* NL1 was analyzed by qRT-PCR. Compared to the control growing on sodium acetate as the sole carbon source, the bacteria grown with phenol showed significantly increased transcription of genes coding for phenol hydroxylase and catechol 1,2-dioxygenase (Figure 4A). Thus, the mRNA level of all the analyzed genes was upregulated by more than 100-fold with phenol compared to cultures without phenol; in particular, the expression of *LSNL_2978*, which encodes a large subunit of phenol hydroxylase containing a biferroic center with the catalytic site, showed the highest increase – by 803.41-fold. The qRT-PCR results suggested that the expression of the seven genes, encoding phenol hydroxylase and catechol 1,2-dioxygenase, was strongly induced by phenol.

To verify the functional activity of the identified genes, we cloned and expressed genes encoding phenol hydroxylase (*LSNL_2975–2980*) and catechol 1,2-dioxygenase (*LSNL_2981*) in *E. coli*. The results indicated that *A. lwoffii* NL1 genes expressed in *E. coli* encoded enzymatically active proteins as evidenced by a decrease in A_340_ and increase in A_260_ values compared to the empty plasmid control group (Figures 4B,C), confirming their nature as phenol hydroxylase and catechol 1,2-dioxygenase, respectively.

### Characteristics of Phenol Degradation in *A. lwoffii* NL1

**FIGURE 5:**
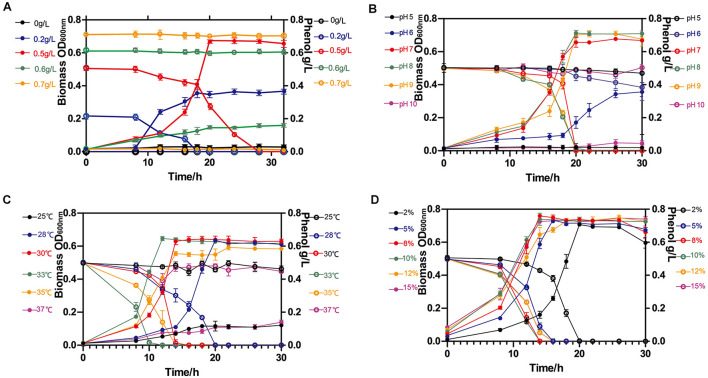
Phenol degradation and cell growth of *A. lwoffii* NL1. **(A)** *A. lwoffii* NL1 growth at different initial phenol concentrations. Effects of initial pH **(B)**, culture temperature **(C)**, and inoculum volume **(D)** on bacterial growth and phenol degradation. Filled circle, biomass OD; hollow circle, phenol concentration. Different conditions were represented by contrasting colors along the ordinate axis.

Cell growth and phenol degradation were monitored in liquid MM medium with phenol as the sole carbon source. The relation between cell growth and phenol degradation generally conforms to a synchronous model. Strain NL1 could metabolize low concentrations of phenol (Figure 5A), but could not grow normally when the concentration equaled or exceeded 0.6 g/L. Thus, the bacteria completely degraded 0.5 g/L phenol in 20 h after a 12-h adjustment period, and their density was close to the maximum level (OD_600_ 0.67).

Then, the effects of pH, temperature, and inoculum volume on phenol degradation by strain NL1 were investigated in 0.5 g/L phenol-containing MM medium. The bacteria could grow at the pH range of 6–9 and completely degrade 0.5 g/L phenol at pH 7–9 within 20 h (Figure 5B). The biomass in the stationary phase was slightly higher at pH 8 or 9 than at pH 7, suggesting that a slightly alkaline environment is more favorable for phenol degradation by *A. lwoffii* NL1. The strain grew well on phenol at the temperature range of 28–35°C (Figure 5C). The optimum temperature for phenol degradation by strain NL1 was 33°C, when its biomass reached the maximum level and phenol was completely degraded in 12 h. At a low proportion of the inoculum (2%), strain NL1 grew on 0.5 g/L phenol for 20 h. When the inoculum was 5%, the bacteria could reach the logarithmic growth phase and degrade phenol in 16 h, and when it was 8% and 10%, phenol was degraded in 14 h (Figure 5D).

### Resistance Characteristics of *A. lwoffii* NL1

The usual habitats of *Acinetobacter* species are water and soil as well as landfills; they can also colonize vegetables and animals. The most representative *Acinetobacter* species is *A. baumannii* (Mendes et al., 2009). Although *A. lwoffii* is a non-baumannii species, some isolates could cause nosocomial infection (Ku et al., 2000). Considering that strain NL1 was isolated near the Chinese Medicine Manufactory, its drug resistance was tested by the paper disc diffusion method according to standard guidelines in Supplementary Table 1. Among the 30 tested antibiotics (Table 3), *A. lwoffii* NL1 showed high resistance to kanamycin, clindamycin, minocycline, ceftazidime, furazolidone, cefradine, cefazolin, selectrin, piperacillin, carbenicillin, medemycin, cefoperazone, and oxacillin, but was sensitive to chloramphenicol, polymyxin B, doxycycline, neomycin, gentamicin, ofloxacin, and amikacin. These results indicate that *A. lwoffii* NL1 is a strain with multiple drug resistance. In addition, cell growth was investigated on LB agars containing different concentrations of tested metal salts. The minimal inhibitory concentrations (mM) of each salt are Hg^2+^(>0.06), Cu^2+^(0.9), Fe^3+^(2.0), Ni^2+^(0.9), Co^2+^(>0.1), Zn^2+^(0.8), and Ag^+^(0.01). The heavy metals resistance give *A. lwoffii* NL1 competitive advantage in wastewater treatment.

Consistent with resistant phenotypes, the whole genome search for drug resistance-related genes performed using the CARD database ((APHA) 2005) and the CGE tools revealed that, in addition to the gene encoding beta-lactamase OXA-10-type located on the chromosome, *A. lwoffii* NL1 carried three genes on pNL1 that encode streptomycin 6-kinase, streptomycin 3′-kinase, and aminoglycoside 3′-phosphotransferase conferring resistance to aminoglycoside antibiotics. The pNL1 as the largest megaplasmid of *A. lwoffii* NL1 contains 345 coding sequences in 18 subsystem categories (Supplementary Figure 3). Among these, genes with metabolic function are mostly involved in the transport and degradation of aromatic compounds (phenol, benzoate, and salicylate) and resistance and transport of heavy metal ions (mercury, ferric iron, copper, and silver).

## Discussion

The distribution of the genes related to phenol catabolic pathway is specific to different microorganisms and even strains. In *A. calcoaceticus* PHEA-2, highly similar catabolic genes are located on the chromosome (Arai et al., 2000; Xu et al., 2003), whereas in *Pseudomonas* sp. strain CF600 and *P. putida* strain H the genes encoding the *meta*-cleavage pathway of phenol degradation are located on plasmids (Herrmann et al., 1995; Powlowski and Shingler, 1994). *A. lwoffii* NL1 degrades phenol via the *ortho*-cleavage pathway, and the genes encoding phenol hydroxylase, catechol 1,2-dioxygenase, and putative regulatory factors are located on the megaplasmid pNL1. The *LSNL_2973* and *LSNL_2983*, located upstream and downstream of phenol catabolic operon, were, respectively, putative transposases for insertion sequence elements IS6501 and IS600 (Figure 3). This indicates that *A. lwoffii* NL1 might acquire phenol degradation pathway-encoding genes by horizontal transfer of mobile genetic elements (Elken et al., 2020). A similar phenomenon of genetic transfer has occurred on mobilizable catabolic plasmids of some *Pseudomonas* strains (Peters et al., 1997; Peters et al., 2004; Wang et al., 2007).

*A*. *lwoffii* NL1 has also been shown to cross resistance to multiple antibiotics and heavy metal ions. Those resistance genes were mostly found on the plasmid pNL1 of *A. lwoffii* NL1. Thus, genes coding for aromatic ring-degrading enzymes and antibiotic resistance are located together on the megaplasmid pNL1, which was also observed in strains isolated from artificial wastewater treatment bioreactors (Xia et al., 2019). The co-occurrence of genes related to pollutant metabolization and drug resistance suggests environmental selection and enrichment; such genes can be potentially to spread *via* mobile elements of certain replicons in polluted wastewater environments (Jiao et al., 2017).

In the present study, a phenol-degrading strain *A*. *lwoffii* NL1 has been shown to tolerate 1.1 g/L phenol and degrade 0.5 g/L phenol within 12 h. The 41.67 mg/L per hour degradation efficiency is higher than that of other reported phenol-degrading strains, such as *P. cepacia* (17.36 mg/L per hour) (Arutchelvan et al., 2005), *R. aetherivorans* (35.7 mg/L per hour) (Nogina et al., 2020), *C. tropicalis* mutants (36.88 mg/L per hour) (Jiang et al., 2007), and so on. *A. lwoffii* NL1 also showed resistance against various heavy metal ions. Thus, the bacteria can be used for the bioremediation of phenol-contaminated industrial wastewaters. In addition, optimized mixed cultures with *A. lwoffii* NL1 would be considered in phenol-contaminated wastewater treatment owing to the greater stability, complete mineralization and increased metabolic capabilities (Sivasubramanian and Namasivayam, 2015).

## Data Availability Statement

The datasets presented in this study can be found in online repositories. The names of the repository/repositories and accession number(s) can be found below: https://www.ncbi.nlm.nih.gov/, CP062199-CP062122.

## Author Contributions

NX, MG, and CY conceived the project. NX, CQ, MW, and MG completed wet experiments. QY and YZ analyzed genome and plasmid. NX, CY, and QY wrote the manuscript. All authors contributed to the article and approved the submitted version.

## Conflict of Interest

The authors declare that the research was conducted in the absence of any commercial or financial relationships that could be construed as a potential conflict of interest.

## Publisher’s Note

All claims expressed in this article are solely those of the authors and do not necessarily represent those of their affiliated organizations, or those of the publisher, the editors and the reviewers. Any product that may be evaluated in this article, or claim that may be made by its manufacturer, is not guaranteed or endorsed by the publisher.

## Footnotes

1
https://tygs.dsmz.de

2
https://cge.cbs.dtu.dk/services/KmerFinder/

3
www-is.biotoul.fr

## Supplementary Material

Supplementary Figure 1 Morphological characteristics of *A. lwoffii NL1*.

Supplementary Figure 2 Phylogenetic trees were constructed based on published 16S rRNA of *Acinetobacter*.

Supplementary Figure 3 Plasmid profile of the pNL1.

Supplementary Table 1 Standard guidelines for antibiotics susceptibility testing of *A. lwoffii NL1*.

Supplementary Table 2 General features of *A. lwoffii* NL1 genome.
