# Supplementary material for: Analysis of Phenol Biodegradation in Antibiotic and Heavy Metal Resistant Acinetobacter lwoffii NL1
Source: Front Microbiol. 2021 Sep 10;12:725755. doi: 10.3389/fmicb.2021.725755 (PMC8461059; doi:10.3389/fmicb.2021.725755)
Supplement: Supplementary Figure 1 — Morphological characteristics of A. lwoffii NL1. [file Data_Sheet_1.docx]

Supplementary Material

Contents of the text:

Supplementary Table 1 (Standard guidelines for antibiotics susceptibility testing of *A. lwoffii* NL1) 2

Supplementary Figure 1 (Morphological characteristics of *A. lwoffii* NL1) 3

Supplementary Figure 2 (Phylogenetic trees were constructed based on published 16S rRNA of *Acinetobacter*) 4

Supplementary Table 2 (General features of *A. lwoffii* NL1 genome) 5

Supplementary Figure 3 (Plasmid profile of the pNL1) 6

Supplementary Table 1. Standard guidelines for antibiotics susceptibility testing of *A. lwoffii* NL1

| Antibiotics | Disk content  (μg) | Interpretive categories and diameter of inhibition zones(mm) | | | Standard guidelines |
| --- | --- | --- | --- | --- | --- |
|  |  | S | I | R |  |
| Chloramphenicol | 30 | ≥18 | 13-17 | ≤12 | WS/T125-1999 |
| Tetracycline | 30 | ≥15 | 12-14 | ≤11 | M100 |
| Kanamycin | 30 | ≥18 | 12-17 | ≤13 | WS/T125-1999 |
| Clindamycin | 2 | ≥21 | 15-20 | ≤14 | Instructions |
| Erythromycin | 15 | ≥23 | 14-22 | ≤13 | Instructions |
| Ceftazidime | 30 | ≥23 | 15-22 | ≤14 | M100 |
| Minocyline | 30 | ≥16 | 13-15 | ≤12 | M100 |
| Cefuroxime | 30 | ≥23 | 15-22 | ≤14 | WS/T125-1999 |
| Doxycycline | 30 | ≥13 | 10-12 | ≤9 | M100 |
| Furazolidone | 300 | ≥17 | 15-16 | ≤14 | Instructions |
| Cefradine | 30 | ≥18 | 13-17 | ≤14 | WS/T125-1999 |
| Cefazolin | 30 | ≥18 | 13-17 | ≤14 | WS/T125-1999 |
| Selectrin | 23.75 | ≥16 | 11-15 | ≤10 | M100 |
| Polymyxin B | 300 | ≥12 | 9-11 | ≤8 | Instructions |
| Neomycin | 30 | ≥17 | 13-16 | ≤12 | Instructions |
| Cefalexin | 30 | ≥18 | 13-17 | ≤14 | WS/T125-1999 |
| Vancomycin | 30 | ≥15 | 11-14 | ≤10 | Instructions |
| Piperacillin | 100 | ≥21 | 18-20 | ≤17 | M100 |
| Ciprofloxacin | 5 | ≥21 | 16-20 | ≤15 | M100 |
| Gentamicin | 10 | ≥15 | 13-14 | ≤12 | M100 |
| Carbenicillin | 100 | ≥23 | 20-22 | ≤19 | WS/T125-1999 |
| Ofloxacin | 5 | ≥17 | 14-16 | ≤13 | WS/T125-1999 |
| Amikacin | 30 | ≥17 | 15-16 | ≤14 | M100 |
| Ampicillin | 10 | ≥15 | 12-14 | ≤11 | WS/T125-1999 |
| Norfloxacin | 10 | ≥17 | 13-16 | ≤12 | WS/T125-1999 |
| Medemycin | 30 | ≥18 | 12-17 | ≤13 | Instructions |
| Cefoperazone | 75 | ≥21 | 16-20 | ≤15 | WS/T125-1999 |
| Oxacillin | 1 | ≥13 | 11-12 | ≤10 | Instructions |
| Ceftriaxone | 30 | ≥21 | 14-20 | ≤13 | WS/T125-1999 |
| Penicillin | 100 | ≥21 | 18-20 | ≤17 | WS/T125-1999 |

The referred M100 and WS/T125-1999 were specific for *Acinetobacter* spp.

The instructions of drug disks meet the standard of antibiotics susceptibility test.


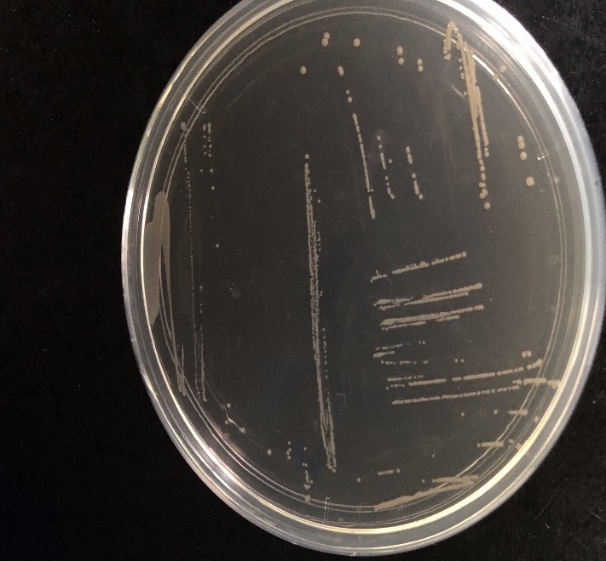

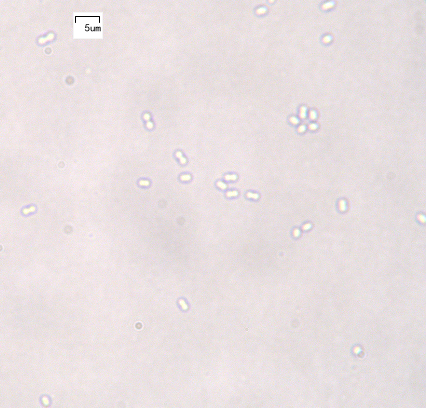


(B)

(A)

Supplementary Figure 1. Morphological characteristics of *A. lwoffii* NL1.

(A) Colonies formed on LB plates after 2 days. (B) Microscopic examination. Colonies formed on LB plates after 2 days.


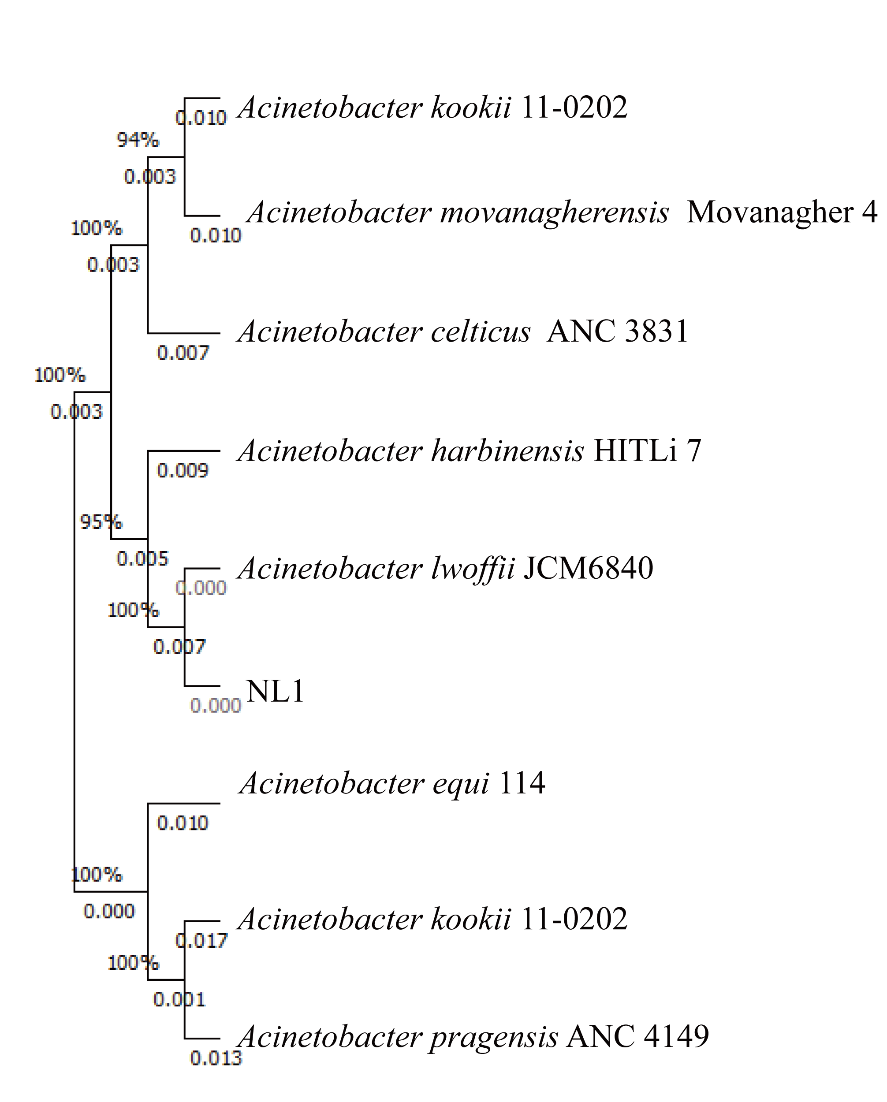


Supplementary Figure 2. Phylogenetic trees were constructed based on published 16S rRNA of *Acinetobacter*.

Supplementary Table 2. General features of *A. lwoffii* NL1 genome.

| Genomic features | |
| --- | --- |
| Genome Size | 3116590 bp |
| G+C content | 43.1% |
| Number of Plasmid | 3 |
| Number of genes | 3499 |
| \| Length of classified repeats (%) \|  \| \| --- \| --- \| | 2.02% |
| Number of rRNA genes | 21 |
| Number of tRNA genes | 86 |


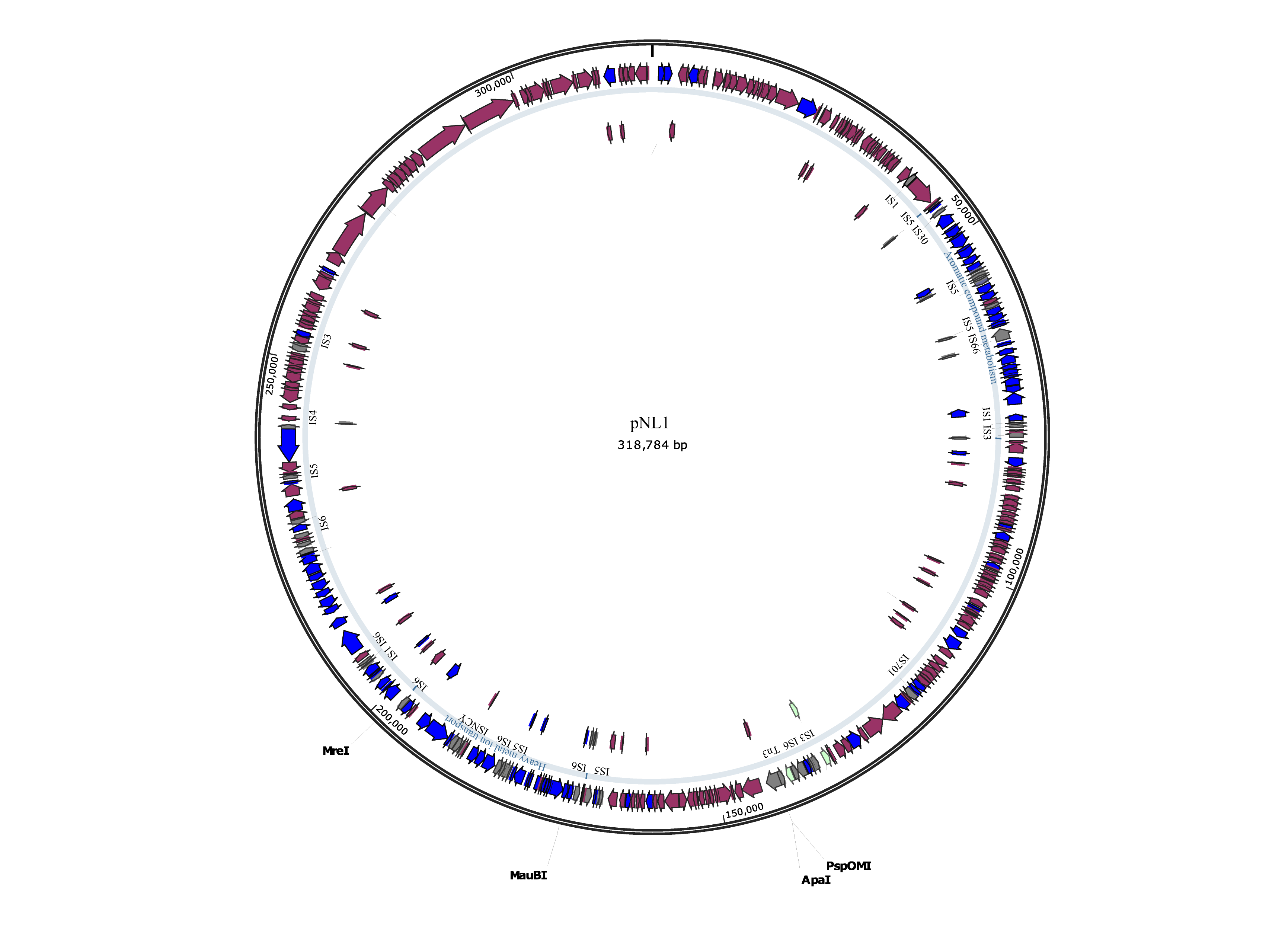


Supplementary Figure 3. Plasmid profile of the pNL1.
